# Compositional characteristics of human peripheral TRBV pseudogene rearrangements

**DOI:** 10.1038/s41598-018-24367-2

**Published:** 2018-04-12

**Authors:** Bin Shi, Long Ma, Xiaoyan He, Peipei Wu, Peng Wang, Xiaomei Wang, Rui Ma, Xinsheng Yao

**Affiliations:** 1 Department of Immunology, Research Center for Medicine & Biology, Innovation & Practice Base for Graduate Students Education, Zunyi Medical University, Zunyi, China; 2 Department of Laboratory Medicine, The first Affiliated Hospital of Zunyi Medical University, Zunyi, China; 3 School of Laboratory Medicine, Zunyi Medical University, Zunyi, China

## Abstract

The diversity of the T cell receptor (TCR) complementarity determining region 3 (CDR3) repertoire is the result of random combinations, insertions and deletions during recombination of the germline V, D and J gene fragments. During evolution, some human TCR beta chain variable (TRBV) pseudogenes have been retained. Many previous studies have focused on functional TRBV genes, while little attention has been given to TRBV pseudogenes. To describe the compositional characteristics of TRBV pseudogene rearrangements, we compared and analysed TRBV pseudogenes, TRBV open reading frames (ORFs) and functional TRBV genes via high-throughput sequencing of DNA obtained from the peripheral blood of 4 healthy volunteers and 4 patients. Our results revealed several differences in J and D gene usage. The V deletion distribution profile of the pseudogenes was significantly different from that of the ORFs and functional genes. In addition, arginine, lysine and cysteine were more frequently used in putative CDR3 pseudogene rearrangements, while functional rearrangements used more leucine. This study presents a comprehensive description of the compositional characteristics of peripheral TRBV pseudogene rearrangements, which will provide a reference for further research on TRBV pseudogenes.

## Introduction

T cell receptor (TCR), which is located in the T lymphocyte membrane, is an important functional receptor that participates in the cellular immunological response. Healthy human individuals usually contain approximately 10^10^ TCRs^1^, and the variety in these TCRs ensures that the level of cellular immunity in the body can adequately respond to a complex antigen environment. TCRs are either TCR αβ or TCR γδ heterodimers, formed from four peptide chains, namely, α, β, γ and δ. Of these, TCR αβ is the main receptor for the cellular immunological response. The antigenic specificity of TCR is primarily determined by the amino acid (AA) sequence of the hypervariable complementarity determining region 3 (CDR3). The nucleotide sequences of CDR3 are generated through random recombination of the segregated germline variable (V), diversity (D), and joining (J) gene segments of the TCRβ chain (TRB) and the V and J gene segments of the TCRα chain (TRA).

As the most widely studied TCRβ chain, the human TCRβ locus consists of 48 functional TRBV genes, 19 TRBV pseudogenes (or ORFs (open reading frames)), 2 functional TRBD genes, 13 functional TRBJ genes and one TRBJ pseudogene. The “pseudogene” identifies the molecule type or the gene functionality in the undefined or germline configuration, which has a stop codon(s) and/or frameshift mutation(s) in its coding region, the mutation of which affects the initiation codon (for a conventional or a V gene). In contrast, the “functional gene” identifies the molecule type or the gene functionality in the undefined or germline configuration, which has an ORF without a stop codon in its coding region and for which there is no described defect in the splicing sites, recombination signals, and/or regulatory elements^2^. “ORF” identifies the molecule type or the functionality in an undefined or germline configuration whose coding region has an ORF but (1) alterations have been described in the splicing sites, recombination signals, and/or regulatory elements (2) and/or changes in the conserved AAs have been suggested by researchers to lead to incorrect folding; (3) and/or the entity is an orphan^2^. Generally, ORF TCR genes could arguably represent an intermediate gene type between P (pseudogene) and F (functional) genes.

TCRβ gene rearrangement occurs in a certain order during maturation of T cells in the thymus. During rearrangement, a D fragment is combined with a J fragment to form a DJ fragment, and then, V and DJ are assembled to form a VDJ fragment (a variable region gene with transcriptional activity). The latter is linked to the constant region gene to form a complete, functional beta chain gene. Once the gene rearrangement is completed, the TCRβ gene begins to be transcribed and expressed, and the T cells continue to differentiate and mature. If the gene rearrangement is successful (unproductive rearrangement) and is unable to express TCRβ, the T cells do not further differentiate, resulting in apoptosis^3^. Usually, the T cell has a second chance to rearrange a functional TCRβ^4^, while the pseudogene cannot encode for productively rearranged receptor genes. Some pseudogenic alleles carry non-functional recombination signal sequences, which prevent their incorporation into rearranged genes^5^.

Currently, the origin of TRBV pseudogenes is unclear. Whether TRBV pseudogenes are involved in rearrangements, can be successfully rearranged or transcribed, or produce functional translation products are topics of ongoing debate. In 1999, Burkhard *et al*. sequenced more than 500 TRBV30, TRBV21-1 and TRBV23-1 genes, and the results showed that there were different CDR3 lengths between the in-frame productive sequences (TRBV30) and in-frame non-productive sequences (TRBV21-1 (pseudogene), TRBV23-1 (ORF)), but there were no significant differences in the J and D gene usages and nucleotide additions to junctional sequences^6^. Meanwhile, Manne *et al*. reported that the TRBV21-1 pseudogene in metastatic melanoma patients appeared to undergo monoclonal expansion^7^. Not long after, Rezvany MR *et al*. found that the TRBV21-1 pseudogene stimulated by leukaemic B cells in CD4^+^ and CD8^+^ T cells showed oligoclonal expansion^8^. These early studies were based on analysis of the intrinsic defects in the TRBV pseudogene sequences or indirectly demonstrated the in-frame and out-of-frame rearrangements of the TRBV pseudogenes and examined whether the TRBV pseudogenes show clonal or oligoclonal mRNA expression under various pathological conditions. Although TRBV pseudogenes cannot be expressed, previous studies have shown that TRBV pseudogenes are still involved in some functional events through unclear mechanisms.

More recently, with the application of high-throughput sequencing techniques to the analysis of the TCR repertoire, it has become possible to compare and analyse the rearrangement characteristics of functional TRBV genes and TRBV pseudogenes in detail from relatively large TCR repertoires^9,10^. Murugan *et al*. reported pseudogene usage using the Illumina high-throughput sequencing approach^11^. Additionally, high-throughput sequencing of the TCRβ locus of a large cohort of healthy volunteers was performed to evaluate the use of functional and non-functional alleles of individual TCRβ V gene segments. This study mainly focused on the annotation of pseudogene segments^12^. However, there has been no systematic analysis of the characteristics of TRBV pseudogenes.

In this study, we broadly analysed TRBV pseudogenes, the TRBV ORFs and functional TRBV genes via high-throughput sequencing of DNA obtained from the peripheral blood of 4 healthy volunteers and 4 unrelated patients. These analyses assessed gene frequency, J and D gene usage, CDR3 AA composition of the in-frame sequence, and nucleotide insertions and deletions. This study will offer insight into the compositional characteristics of peripheral TRBV pseudogene rearrangements.

## Results

### Gene frequency

**Figure 1:**
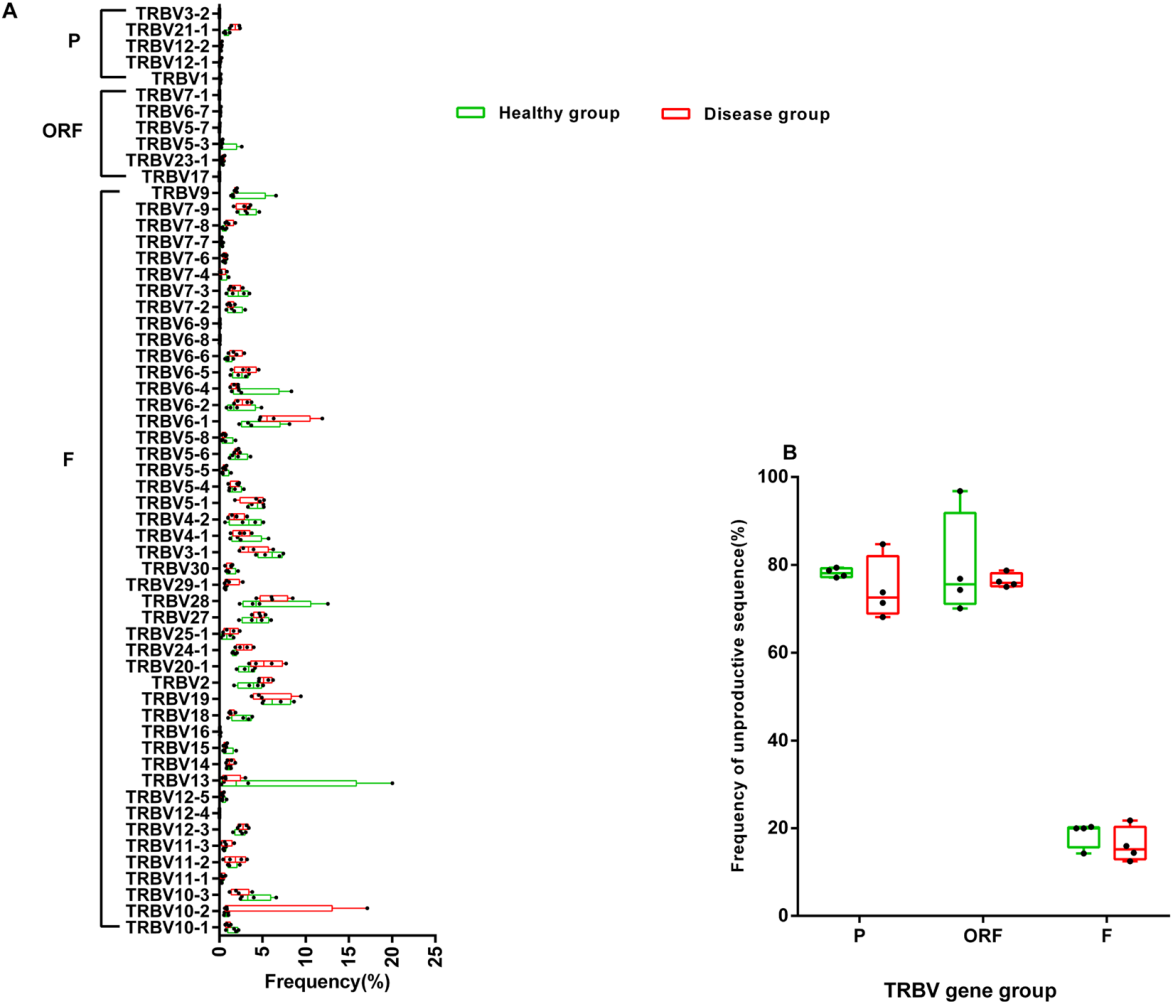
Gene frequency of the TRBV functional (F) genes, TRBV open reading frames (ORFs) and TRBV pseudogenes (P) in the healthy and disease groups. (**A**) The frequency of functional TRBV genes is significantly higher than that of the TRBV pseudogenes and ORFs. (**B**) TRBV pseudogenes and ORFs show unproductive rearrangements that are significantly used, while most functional genes are productive. These results are plotted with a box line diagram, which shows all the points to indicate each sample. The line indicates the median, and two error bars are used to represent the min and max.

The list of sequence data from 4 healthy samples and 4 patients is shown in Table 1. The sequences with No results or Unknown or V-region identity <85% were filtered out. We finally analysed all the TRBV functional (F) rearrangements, TRBV ORF rearrangements and TRBV pseudogene (P) rearrangements (Table 1). First, we investigated the frequencies of these three classes of rearrangements (Fig. 1A,B). Considering the sample size and some extreme values, we used the median to evaluate these data. Figure 1A shows that the frequencies of TRBV P and ORF rearrangements (median 1.0% and 0.7% in the healthy group; median 2.3% and 0.9% in the disease group) were much lower than that of the F rearrangements (median 85.6% in the healthy group and median 88.7% in the disease group). In the healthy group, the frequency of the TRBV21-1 pseudogene was higher than the sum of the frequencies of the other pseudogenes (0.7% VS 0.3%), while other pseudogenes were not detected due to the low frequency (functional gene subset covered 45 genes). This disparity was enlarged in the disease group due to the expansion of TRBV21-1 (1.9% VS 0.4%), which may also be related to the disease condition. Different from P and ORF rearrangements, there are obviously different usage of the F rearrangement between the healthy and disease groups (Fig. 1A). Figure 1B shows that F rearrangements are mostly productive rearrangements, while P and ORF rearrangements are characterized by a high proportion of unproductive rearrangements. Taken together, these results suggested that the TRBV pseudogene rearrangements and functional gene rearrangements may have significant differences in composition and prompted us to compare their compositional characteristics.

**Table 1:** Number of sequences in different categories identified in 4 healthy individuals and 4 patients.

Donors	Raw sequences	No results	Unknown	V-region identity < 85%	ORF	F	P
H1	384,953(22008)	165(17)	4,458(322)	424(27)	3,427(283)	370,024(20690)	6,358(648)
H2	405,628(22535)	75(13)	1,725(284)	184(15)	2,174(269)	397,731(21384)	3,576(547)
H3	609,445(39294)	107(12)	2,895(485)	297(43)	3,643(461)	596,657(37393)	5,708(866)
H4	546,293(16113)	67(7)	1,962(205)	140(19)	16,971(199)	521,783(15296)	5,228(372)
D1	1,722,089(19935)	582(25)	5,587(122)	445(26)	13,506(299)	1674,216(18902)	27,753(561)
D2	1481,440(42069)	592(34)	7,260(219)	831(38)	14,375(572)	1415,909(40078)	42,473(1128)
D3	884,760(43599)	388(26)	4,646(252)	332(31)	6,418(409)	849,370(41973)	23606(908)
D4	1219,887(32372)	1,760(29)	6,558(173)	824(36)	14,208(416)	1172,959(30982)	23,578(736)

Note: H1-4 represent healthy individuals and D1-4 represent patients; data in brackets is unique.

### J and D gene usage

**Figure 2:**
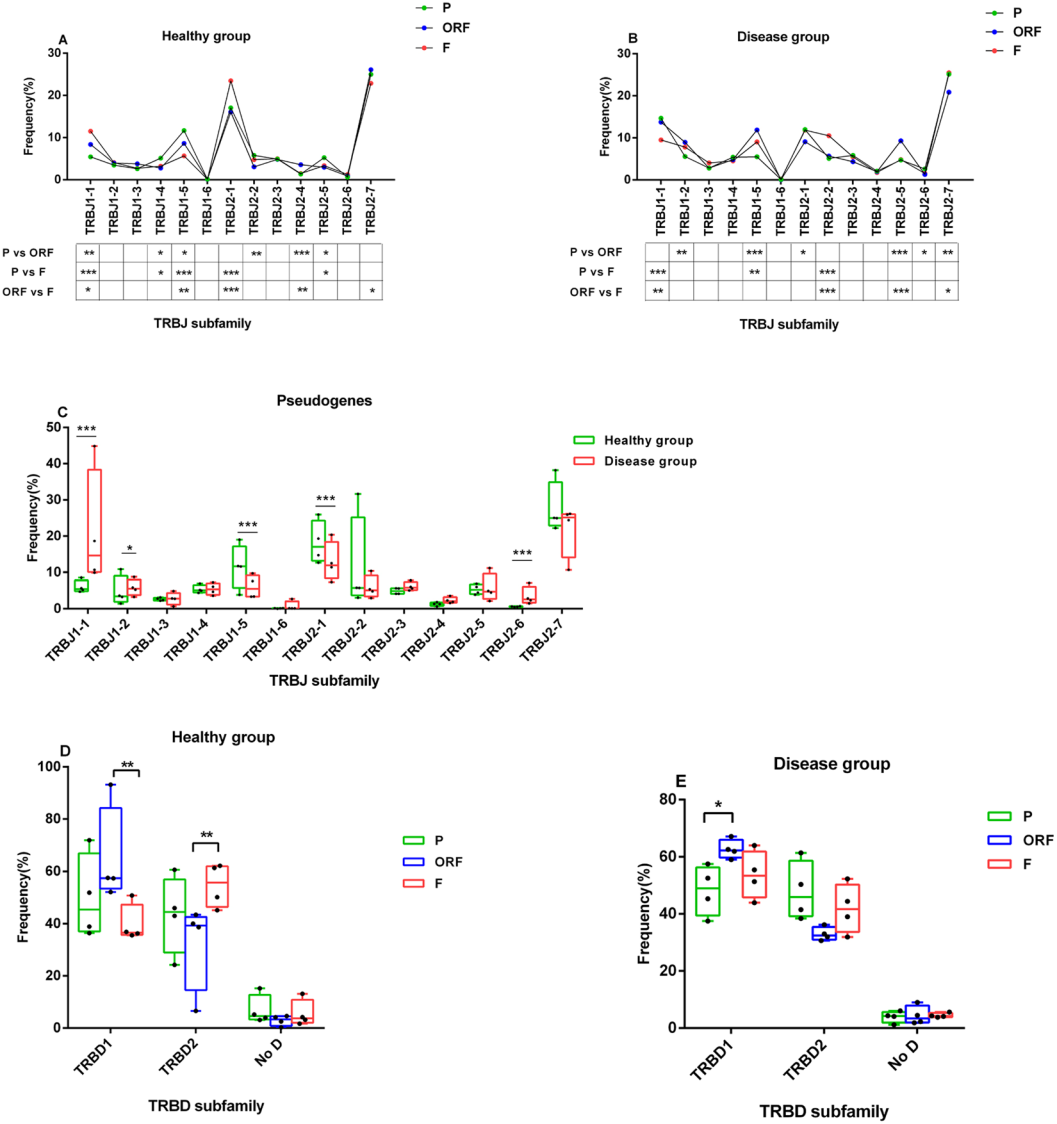
Usage of TRBJ and TRBD genes in 4 healthy individuals and 4 patients. (**A**,**B**) Usage of the TRBJ genes show significant differences among three classes of rearrangements in the healthy and disease groups. The results are shown with the median value in each group. The p values were determined with a χ2 test using the median of four samples in each group. All statistically significant differences are indicated. *p < 0.05, **p < 0.01, ***p < 0.001. (**C**) Comparison of the TRBJ usage in the pseudogene between the healthy and disease group is shown. (**D**,**E**) Usage of TRBD genes in the three classes of rearrangements in the healthy and disease groups is shown. The results (**C**–**E**) are plotted with a box line diagram, which shows all the points to indicate each sample in each group. The line indicates the median, and the two error bars represent the min and max. The p values were determined with a χ2 test using the median of four samples in each group. All statistically significant differences are indicated. *p < 0.05, **p < 0.01, ***p < 0.001.

The human TCRB gene locus contains 13 TRBJ segments and 2 TRBD segments (No D represents few V-J rearrangements without the TRBD segment). TRBJ usage has been demonstrated to benon-random in productive transcripts derived from peripheral blood T cells^6^. We compared the J and D gene usage of P, ORF and F rearrangements in the healthy and disease groups (Fig. 2A–E). In the healthy and disease groups, we observed significant differences in the overall TRBJ usage among P, ORF and F rearrangements (χ2 test, 12 degrees of freedom, each p < 0.001), whereas the usages of TRBJ1-3, TRBJ1-6 and TRBJ 2-3 were very similar (Fig. 2A,B). In addition, TRBJ2-7 was used at a relatively high frequency (each percentage > 20%) among P, ORF and F rearrangements in the healthy and disease groups. Notably, for the healthy group, the usage frequency of TRBJ1-1 in P rearrangements was significantly lower than that in the ORF and F rearrangements (χ2 test, each p < 0.01), and the P rearrangement showed more frequent utilization of TRBJ1-5 than the ORF and F rearrangements (χ2 test, p < 0.05 and p < 0.01, respectively). Additionally, for the disease group, the usage frequency of TRBJ1-5 in P rearrangements was significantly lower than that in the ORF and F rearrangements (χ2 test, each p < 0.01) (Fig. 2A,B). We also found several differences in the TRBJ usage of P rearrangements between the healthy and disease groups, which were embodied in TRBJ1-1, TRBJ1-2, TRBJ1-5, TRBJ2-1 and TRBJ2-6 (Fig. 2C). In addition, the TRBD1 and TRBD2 usages of the P rearrangements were relatively equal in the healthy and disease groups (45.3% vs 44.4% and 48.9% vs 45.9%, respectively), while nonconformity in the differences in TRBD1 or TRBD2 usage appeared between the healthy and disease groups (Fig. 2D,E). Although there were several differences among the P, ORF and F rearrangements, it was difficult to distinguish the P rearrangements based on the frequencies of the J or D gene.

### Presumptive CDR3 AA composition of in-frame rearrangement

**Figure 3:**
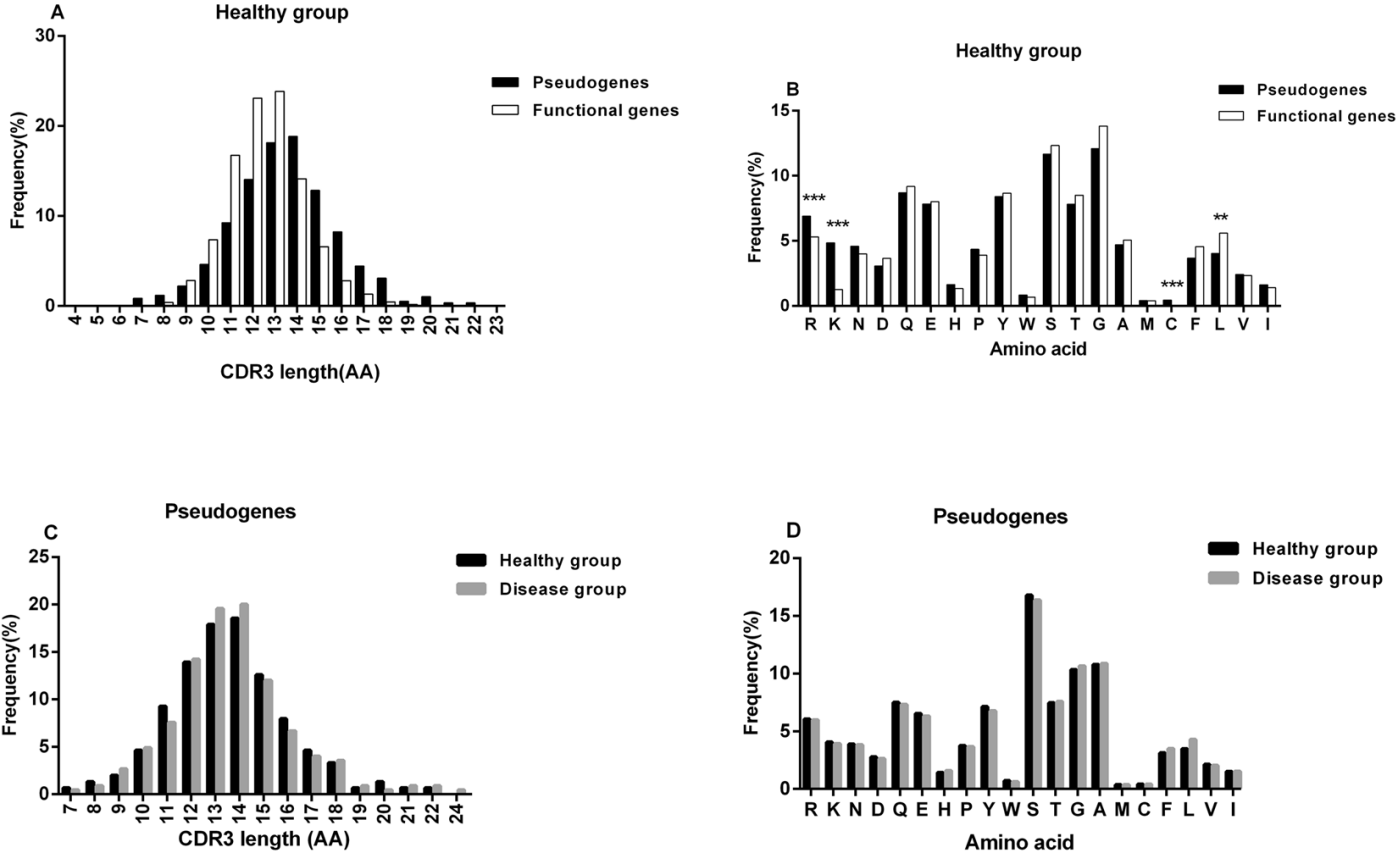
CDR3 lengths and overall amino acid usage of the CDR3 regions in in-frame functional TRBV gene rearrangements and in-frame TRBV pseudogene rearrangements. (**A**) Average CDR3 length distribution of in-frame TRBV functional gene rearrangements and in-frame TRBV pseudogene rearrangements in the healthy group are shown. (**B**) The amino acids are arranged by relative hydrophobicity values according to the Kyte-Doolittle scale^13^. The overall CDR3 amino acid composition differed significantly between the pseudogene rearrangements and functional gene rearrangements in the healthy group (χ2 test, p < 0.001). (**C**,**D**) Comparison of the average CDR3 length distribution and the overall CDR3 amino acid composition of the in-frame TRBV pseudogene rearrangements between the healthy and disease group shows no differences. The results are shown as the mean value in each group. The p values were determined using a χ2 test (**B** and **D**). All statistically significant differences are indicated. *p < 0.05, **p < 0.01, ***p < 0.001.

The AA sequence encoded by the V-D-J joining region is obviously heavily influenced by the utilized TRBD and TRBJ germline sequences^6^. Considering the influence of disease, we compared the CDR3 regions between in-frame pseudogene rearrangements and in-frame functional gene rearrangements in the healthy group. The number of unique CDR3 sequences in each sample is shown in Table 2. In the healthy group, we found that the average length of CDR3 in the TRBV pseudogene rearrangements (13.5 ± 2.4 AA residues) was slightly longer than that in the functional gene rearrangements (12.5 ± 1.8 AA residues) (Fig. 3A). Overall, the AA usage in these unique CDR3 sequences showed more frequent usage of arginine, lysine and cysteine in the TRBV pseudogene rearrangements (χ2 test, each p < 0.001), while more leucine was found in functional gene rearrangements (χ2 test, p < 0.01) (Fig. 3B). The usage frequencies of the other AAs were similar between these two groups (Fig. 3B). Interestingly, the CDR3 length distribution and overall AA usage of in-frame pseudogene rearrangements between the healthy and disease group were very similar (Fig. 3C,D).

**Table 2:** Number of unique CDR3 sequences from 4 healthy individuals.

Donors	TRBV functional genes	TRBV pseudogenes
H1	15,588	133
H2	16,385	135
H3	28,947	227
H4	11,522	89

**Figure 4:**
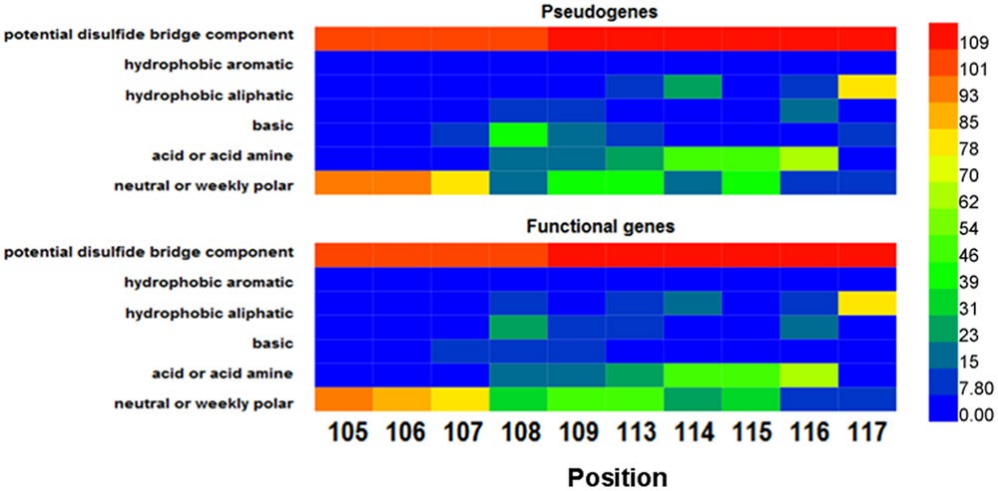
Amino acid frequencies at the ten specific positions selected for assessment of in-frame functional TRBV gene rearrangements in comparison with the putative amino acid utilization in in-frame TRBV pseudogene rearrangements grouped according to physical properties. CDR3 positions (105–117) are shown according to the IMGT unique numbering. The results are plotted with a heat map, which shows the mean in 4 healthy individuals of all the unique productive CDR3 sequences with different lengths.

When AAs were grouped according to their physical properties, the comparison of the functional gene (selected) and pseudogene (non-selected) rearrangements revealed that the pseudogene sequences coded more frequently for basic (position 108) and potential disulphide bridge component residues (position 108 and 109) (χ2 test, each p < 0.001, Fig. 4). IMGT unique numbering is used for position identification^14–16^.

Moreover, we calculated the overall AA frequencies for 10 positions in the CDR3 sequences (Fig. S1). Positions 105, 106, 107, 116 and 117 were considered to be evolutionarily conserved. Interestingly, significant differences in positions 105 and 106 were observed between pseudogene rearrangements and functional gene rearrangements. In position 105, more pseudogene CDR3 used threonine (9.2% VS 0.03%), while more frequent usage of serine was observed in the CDR3 functional gene (12.2% vs 0%). In position 106, arginine and serine were more frequently used in the TRBV pseudogene rearrangements (2.9% VS 0.4% and 93.2% VS 76.8%, respectively), while tryptophan, threonine, alanine and valine were less frequently used.

### Insertion and deletion of nucleotides

The CDR3 diversity results from the addition of “N” nucleotides at the V → D (N1) and D → J (N2) junctions, exonuclease trimming (3′V trimmed, 5′D trimmed and 5′J trimmed) and the addition of palindromic “P” nucleotides (P3′V, P5′D and P5′J). Considering the functionality of the sequence, we compared insertions and deletions between the total TRBV pseudogene rearrangements and the TRBV functional gene rearrangements in the healthy group (Fig. S2). We found that the average number of nucleotides added at N1 or N2 in the TRBV pseudogene rearrangements was more than 1 nt greater than that added in the TRBV functional gene rearrangements (ANOVA with Bonferroni correction, p < 0.001), while no significant difference in the usage of the four bases at N1 or N2 was observed between the TRBV pseudogene rearrangements and functional TRBV gene rearrangements (Fig. S2). There were similar additions in the P regions (P3′V, P5′D and P5′J) and deletions at 3′V, 5′D and 5′J by exonuclease trimming (Fig. S2). Furthermore, we removed the background noise of the sequence to analyse unique sequences in the three classes of rearrangements. Due to the different distribution profiles of the nucleotide insertions or deletions in different functional TRBV or TRBJ genes^11^, the proportion of TRBV21-1 detected in the pseudogene group was relatively high; therefore, we observed the N insertion profiles of TRBV21-1 and no-TRBV21-1 (Fig. S3). Notably, similar to the functional genes, the different TRBV pseudogenes exhibited different insertion profiles (Fig. S3).

**Figure 5:**
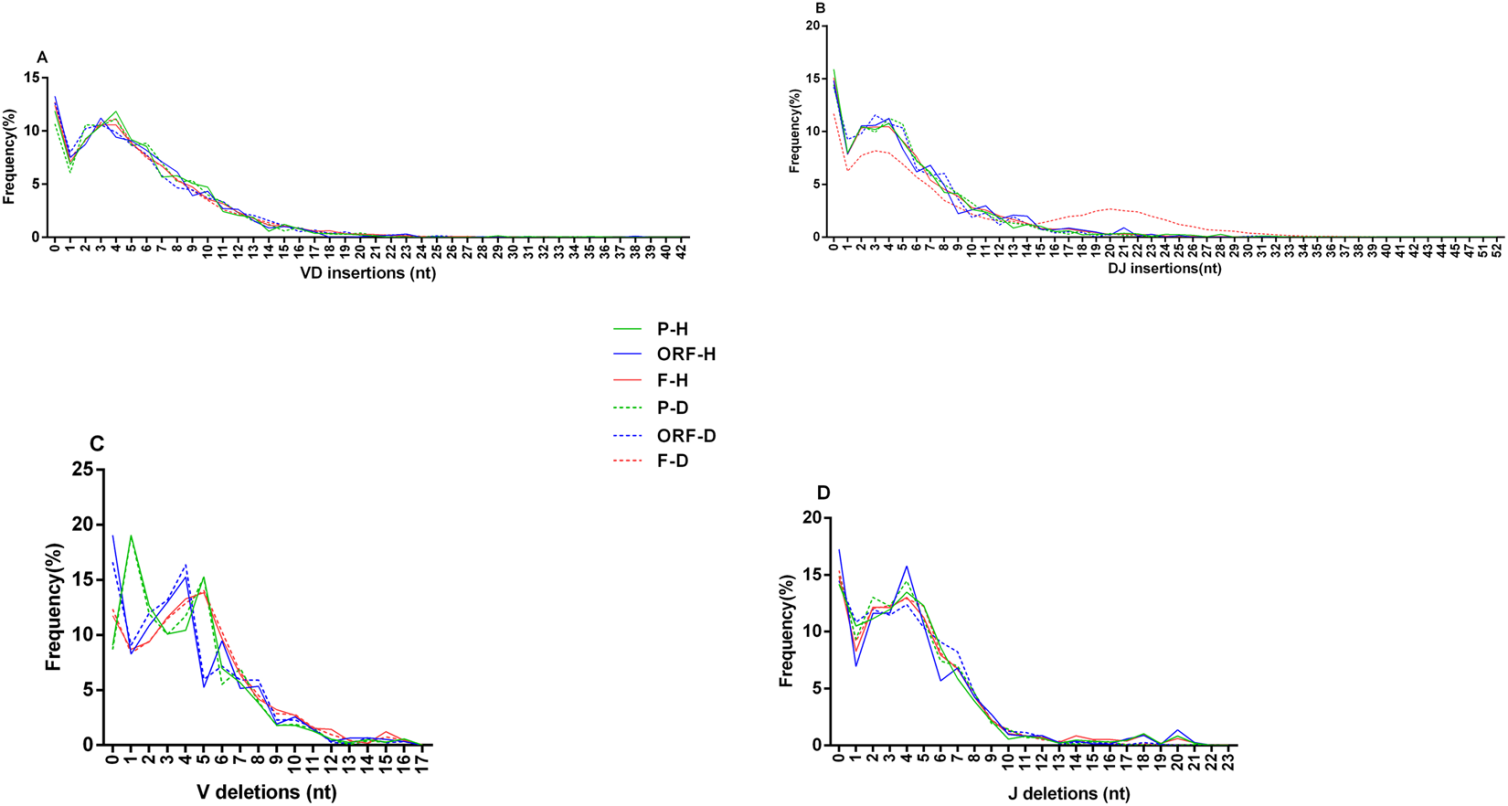
Insertions and deletions of the nucleotides in V-D-J junctions of P, ORF, and F rearrangements in the healthy (P-H, ORF-H and F-H) and disease group (P-D, ORF-D and F-D). (**A**) The frequency profiles of the VD insertions of different nucleotide lengths in three classes of rearrangements. (**B**) The frequency profile of the DJ insertions of different nucleotide lengths in three classes of rearrangements. (**C**) The frequency profile of the V deletions of different nucleotide lengths in three classes of rearrangements. (**D**) The frequency profile of the J deletions of different nucleotide lengths in three classes of rearrangements. The results are shown as the mean of all unique unproductive sequences in each group.

According to the classification method for nucleotide insertion and deletion reported by Murugan *et al*.^11^, we investigated the nucleotide distribution profiles among the pseudogenes, ORFs and functional genes (Fig. 5A,D).

The VD insertion profile was similar among the P, ORF and F genes in both the healthy and disease group (Fig. 5A). The DJ insertion profile of the F rearrangements deviated from those of other groups, which may be related to disease (Fig. 5B). Notably, the V deletion profiles of the nucleotides among the P, ORF and F genes were obviously different in both the healthy and disease group (Fig. 5C). In addition, the V deletion profiles of the same type of genes were similar between the healthy and disease group. Therefore, the characteristics of the V deletion profile of nucleotides could distinguish the pseudogene repertoire from the ORF and F repertoires. Here, the characteristics of the V deletion profile of pseudogenes mainly reflected the high proportion of short nucleotide deletions (1 nt) and the high incidence rate of nucleotide deletions (more than 90%).

## Discussion

The diversity of the TCR CDR3 repertoire is the result of random combinations, insertions and deletions during rearrangements of the germline V, D and J gene fragments. During evolution, some human TRBV pseudogenes have been retained. Past studies have only compared single pseudogenes with single functional genes. In addition, due to the limited number of analysable sequences and methodology limitations, it was previously difficult to accurately describe the compositional characteristics of TRBV pseudogenes. Despite recent analysis of a large sample (587 healthy individuals) that focused on annotation of pseudogene segments through massive parallel sequencing^12^, the detailed compositional characteristics of TRBV pseudogenes still need to be elucidated. In a way, ORFs can be seen as a special group between the pseudogene and functional gene. In addition, the TRBV pseudogene should be less affected by disease. Thus, we performed broad analyses of the human peripheral TRBV pseudogene, TRBV ORF and functional gene rearrangements in heathy and disease conditions using high-throughput sequencing.

Usually, in the thymus, a functional TRBV gene combines randomly with a TRBJ and a TRBD to form a V-D-J rearrangement at the pre-T stage. After selection events, the TRBV functional gene rearrangement will be expressed in the periphery, participating in the cellular immunological response^11,17^. However, TRBV pseudogene rearrangement is non-selective, and these transcripts will not be expressed in the periphery. Therefore, the frequency of TRBV pseudogene rearrangement we observed was much lower than that of the functional rearrangements. We observed that usage frequencies in the TRBV pseudogenes (less than 2%) were lower than previously reported^11^. Previous results showed significant usage of TRBV23-1, TRBV5-3, TRBV12-2 and TRBV6-7 (together accounting for almost 10% of CDR3 sequence reads)^11^. These differences were associated with different sequencing methods, filtering criteria (this paper was based on the IMGT criteria, and TRBV23-1 was divided into ORF groups) and cell subsets or sample sources. Similarly, these factors led to differences in usage frequencies of functional genes between this study and previous reports^9–11^.

Due to the high proportion of unproductive rearrangements in the TRBV pseudogene and ORF, pseudogene characteristics should be identified based on unproductive sequences. When we investigated J and D gene usage, several differences in J and D gene usage were observed among the TRBV pseudogene, ORF and functional gene groups, while it was difficult to distinguish the pseudogene based on the frequencies of the J or D gene. Although analysis of all the data showed that TRBV pseudogenes had N1 and N2 insertions of more than 1 nt compared with the functional genes, analysis of the unique data showed that there were no obvious differences in the length of the insertion or deletion among these three types of rearrangements. Notably, similar to the functional genes^11^, there were different insertion profiles in the different TRBV pseudogenes. Further analyses suggested that the V deletion distribution profile of the pseudogenes was obviously different from that of the functional genes and the ORF. Thus, the characteristics of the V deletion profile of the nucleotides could distinguish the pseudogene repertoire from the ORF and functional repertoire.

In addition, arginine, lysine and cysteine residues were more frequently used in the putative CDR3 of pseudogene rearrangements, while the functional rearrangement used the leucine residue more. Notably, Chakraborty *et al*. used mathematical modelling to show that the preferentially selected TCRs contain no or few charged AAs, providing strong interaction with the major histocompatibility complex (MHC)^18^. The CDR3 AAs found in the pseudogenes typically contained these AAs, while they were relatively rare in the selected repertoire, confirming the prediction of Chakraborty *et al*.^18^. Moreover, we observed significant differences in the CDR3 AA usage at the conserved 105 and 106 sites, which may be related to evolution. Although the pseudogenes could not be expressed, a small number of productive pseudogene rearrangements containing a CDR3 domain were detected, which could only be observed as the second allele in cells that also expressed a functional TCRβ^3^. Therefore, analysing this presumed domain might help to understand the compositional characteristics of the TRBV pseudogene.

In conclusion, we performed high-throughput sequencing and IMGT/HighV-QUEST analysis of the human peripheral cell repertoire to analyse TRBV pseudogene rearrangement, ORFs and functional rearrangements. The results suggest that the compositional characteristics of TRBV pseudogene rearrangements are different from those of functional TRBV rearrangements, including in gene usage, nucleotide insertion and deletion, and CDR3 composition. These findings provide a reference for further research on TRBV pseudogenes. Because TCRs in memory cells can be skewed as a consequence of the personal history of antigen exposure, further analyses of TCR repertoires in naive and memory T cell subsets would be meaningful. In addition, the TCR alpha chain, which pairs with the TCR beta chain, should be the focus of future research.

## Methods

### Subjects and sample preparation

Peripheral blood was obtained from four unrelated healthy Chinese individuals 25–35 years of age (H1, H2, H3 and H4) and four unrelated Chinese patients 20–36 years of age (2 SLE patients (D1 and D2) and 2 breast cancer patients (D3 and D4)) from the First Affiliated Hospital of Zunyi Medical University. All subjects gave informed consent prior to inclusion in the study. All the research protocols were approved by The Committee on the Ethics of Human Experiments of Zunyi Medical University, and all the experiments were performed in accordance with the guidelines of the committee. Mononuclear cells were obtained from peripheral blood (all donors) using Ficoll 1640 (Biochrom AG, Berlin, Germany) density centrifugation. Total genomic DNA was isolated from each tube of cells using a QIAamp DNA MiniKit (Qiagen, CA) following the manufacturer’s instructions.

### High-throughput sequencing

For each sample, TCRB CDR3 regions were amplified and sequenced from 2 µg of genomic DNA, or if there was less than 2 µg, all the available extracted DNA. Multiplexed PCR amplification was performed to amplify the rearranged CDR3 sequences using mixture of 60 forward primers specific to TCR Vβ gene segments and 13 reverse primers specific to TCR Jβ gene segments^19^. Bias in this multiplexed PCR assay was controlled using synthetic templates^19^. Amplification and sequencing of the TCRB CDR3 regions was carried out using the ImmunoSEQ platform at Adaptive Biotechnologies (http://www.adaptivebiotech.com). The TCRB CDR3 regions were identified within the sequencing reads according to the definition established by the International ImMunoGeneTics (IMGT) collaboration^20^. A standard algorithm was used to identify which V, D, and J segments contributed to each TCRB CDR3 sequence^13,21^.

### Sequence analysis

Raw sequences in the FASTA format were submitted to IMGT/HighV-QUEST online software (http://www.imgt.org). Using the IMGT summary document, the sequences contained the following principles that would be filtered out: (1) No results (sequences for which IMGT/HighV-QUEST did not return any result); (2) Unknown (sequences for which no functionality was detected. This category corresponds to the sequences for which the junction could not be identified (no evidence of rearrangement, no evidence of junction anchors.)); (3) V-region identity < 85%. Sequences after filtering were used for gene frequency, TRBJ and TRBD usage, and nucleotide insertion and deletion analyses. For comparison of the CDR3 AA sequences, CDR3 sequences with out-of-frame rearrangements, stop codons or a CDR3 AA junction lacking a 5′cysteine and 3′phenylalanine were discarded. The proportional usage of the individual AAs was calculated and grouped according to the properties of their side chains, as follows: potential disulphide bridge components (C); hydrophobic aromatics (F, W, Y); hydrophobic aliphatics (L, I, V, M); basic nucleotides (H, K, R); acid or acid amines (Q, N, E, D); and neutral or weekly polar but not charged nucleotides (P, A, G, S, T).

### Software and statistics

IMGT/HighV-QUEST (version 1.3.1) was used for identification of sequences (Vβ, Dβ, Jβ and CDR3), evaluation of functionality and statistical analysis of the sequence data; IMGT/V-QUEST (version 3.3.1) was used for identification of CDR3 positions; Microsoft Office Excel (version 2010) was used for storage, filtering and statistical calculation of the sequences. The resulting sequences were graphed using Prism 6 software (GraphPad). J gene and D gene usage and CDR3 AA were compared using a χ2 test, and insertions and deletions of the nucleotides were compared using two-way ANOVA with Bonferroni correction. p < 0.05 was considered statistically significant.

## Electronic supplementary material

Supplementary Figures
