# Supplementary material for: Compositional characteristics of human peripheral TRBV pseudogene rearrangements
Source: Sci Rep. 2018 Apr 12;8:5926. doi: 10.1038/s41598-018-24367-2 (PMC5897323; doi:10.1038/s41598-018-24367-2)

**Compositional characteristics of human peripheral TRBV pseudogene rearrangements**

**Bin Shi^2,3*^, Long Ma^1,*^, Xiaoyan He^1^, Peipei Wu^3^, Peng Wang^1^, Xiaomei Wang^1^, Rui Ma^1^ & Xinsheng Yao^1^**

^1^Department of Immunology, Research Center for Medicine & Biology, Innovation & Practice Base for Graduate Students Education, Zunyi Medical University, Zunyi, China. ^2^Department of Laboratory Medicine, The first Affiliated Hospital of Zunyi Medical University, Zunyi, China. ^3^School of Laboratory Medicine, Zunyi Medical University, Zunyi, China. *These authors contributed equally to this work. Correspondence and requests for materials should be addressed to X.Y. (email: immunology01@126.com)

**Figure S1.** The frequency of the individual amino acids at the ten specific positions for in-frame functional TRBV gene rearrangements and in-frame TRBV pseudogene rearrangements for CDR3. The colour menu for the amino acids is according to IMGT. CDR3 positions (105-117) are shown according to the IMGT unique numbering. **(A)** TRBV pseudogene rearrangements. **(B)** Functional TRBV gene rearrangements. The results are shown as the mean of all the unique productive CDR3 sequences with different lengths in 4 healthy individuals.


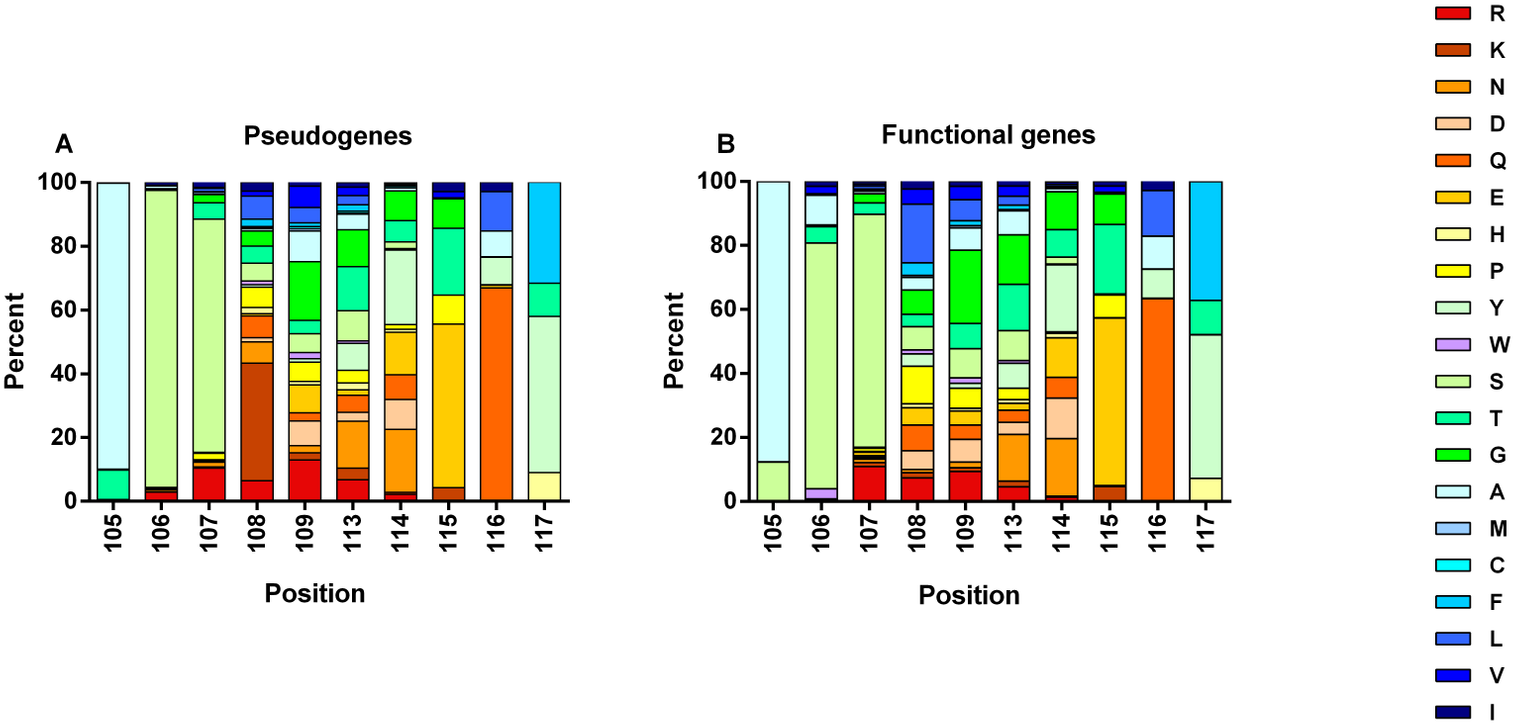


**Figure S2.** Addition and exonuclease trimming of the nucleotides in total sequences from 4 healthy individuals. **(A)** The number of N1, N2, P3'V, P5'D and P5'J nucleotides added at the V-D-J junctions and the nucleotides deleted at 3'V, 5'D, 3'D and 5'J by exonuclease trimming are shown (mean ± SD; error bars represent SD). The p values were determined using two-way ANOVA with a Bonferroni correction. All the statistically significant differences are indicated. * = p < 0.05, ** = p < 0.01, *** = p < 0.001. **(B)** No significant difference in the usage of the four bases at N1 or N2 were found between TRBV functional gene rearrangements and TRBV pseudogene rearrangements.


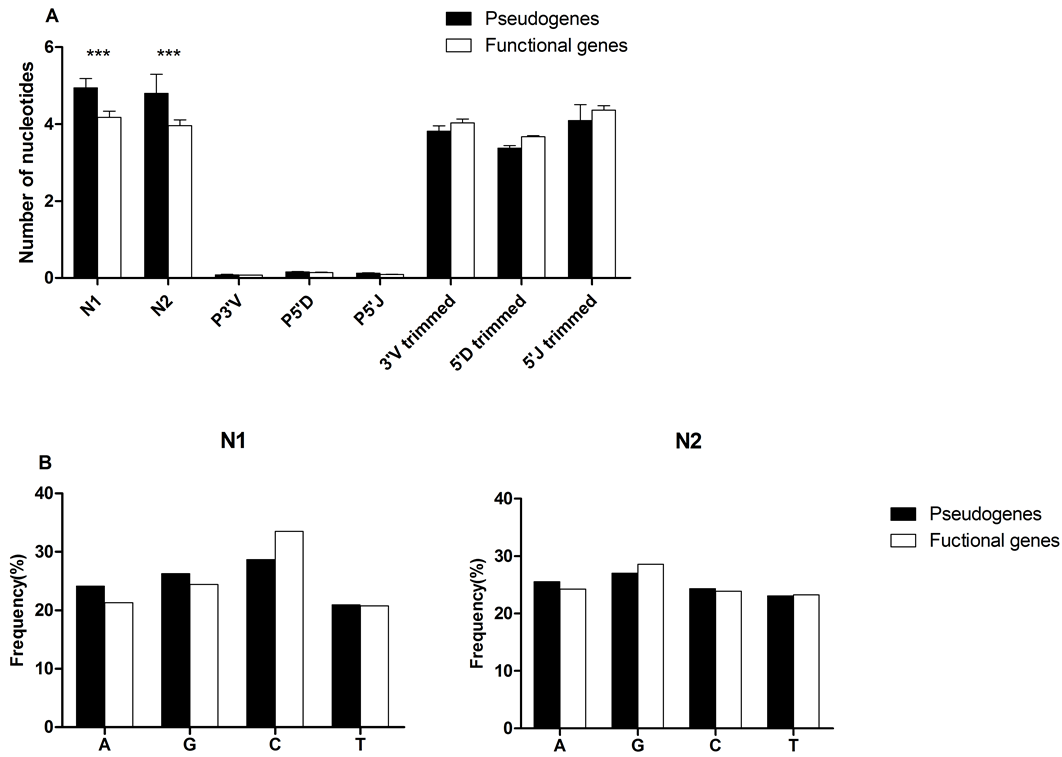


**Figure S3.** Frequency profiles of the N insertions of TRBV21-1 and non-TRBV21-1 in the pseudogene group in each healthy individual. **(A-D)** H1, H2, H3 and H4, respectively. The results are plotted using unique unproductive sequences.


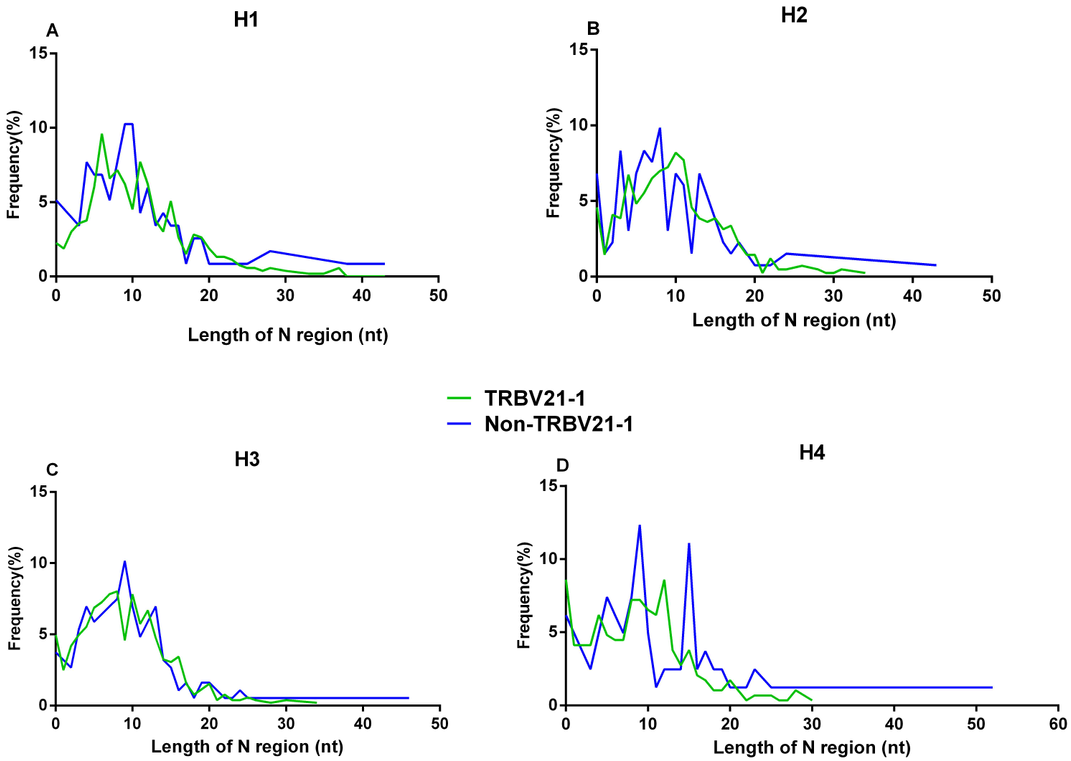

Supplement: Supplementary file 1 — Supplementary Figures [file 41598_2018_24367_MOESM1_ESM.docx]
